# Bidirectional Ventricular Tachycardia: A Hallmark of Catecholaminergic Polymorphic Ventricular Tachycardia

**DOI:** 10.1016/s0972-6292(16)30481-8

**Published:** 2012-04-30

**Authors:** Francisco Femenia, Raimundo Barbosa-Barros, Stela Vitorino Sampaio, Mauricio Arce, Andres Perez-Riera, Adrian Baranchuk

**Affiliations:** 1 Arrhythmia Unit. Cardiology Department. Hospital Espanol de Mendoza. Argentina; 2 Coronary Center. Hospital de Messejana "Dr. Carlos Alberto Studart Gomes". Fortaleza, Ceara. Brazil; 3 Faculdade de Medicina do ABC. Fundacao do ABC. Santo Andre, Sao Paulo. Brazil; 4 Heart Rhythm Service. Queen's University. Kingston, Ontario. Canada

**Keywords:** bidirectional tachycardia, ventricular tachycardia, catecholaminergic polymorphic ventricular tachycardia, syncope, sudden cardiac death

## Abstract

Catecholaminergic polymorphic ventricular tachycardia is a familial cardiac arrhythmia that is related to *RYR2* or *CASQ2* gene mutation. It occurs in patients with structurally normal heart and causes exercise-emotion triggered syncope and sudden cardiac death. We present a 13 year-old girl with recurrent episodes of exercise-related syncope and prior history of sudden death in a first degree relative.

A 13-year-old girl with no cardiovascular history presented for evaluation of recurrent episodes of exercise-related syncope and prior history of sudden death in a first degree relative. The 12- lead electrocardiogram (ECG) at rest was normal and an echocardiogram confirmed a structurally normal heart. A 24-hour Holter monitoring (Figure 1, left panel) during physical activity showed the classic electrocardiographic manifestation of catecholaminergic polymorphic ventricular tachycardia (CPVT): bidirectional ventricular tachycardia (BVT), two alternating QRS complexes morphologies with different polarity (X and Y, Figure 1 right panel) with an XY interval of 240 ms and YX interval of 280 ms. It may be necessary to conduct a 12-lead ECG to confirm if those polarities represented right bundle branch block and left bundle branch block respectively. The BVT presented regular X-X and Y-Y intervals both at 520 ms. According to the classification originally proposed by Scherf and Kisch, the BVT presented here is a Type II BVT, defined by a regular and fixed alternation of short and long intervals.[1,2]

This tachycardia suddenly degenerated into self-limiting polymorphic ventricular tachycardia and ventricular fibrillation (Figure 1, left panel), and normal sinus rhythm resumed upon spontaneous cessation of ventricular fibrillation. The resting 12-lead ECG was normal after the event. (Figure 2)

The patient was treated with propanolol 120 mg/day. During a control exercise stress test the patient presented again with CPVT symptomatic by syncope. An implantable automatic cardioverter defibrillator was implanted (PRIZM DR, Guidant, MN, USA). Genetic screening was not performed at the time of writing this report.

During a ten year follow-up, the patient presented with three episodes of polymorphic ventricular tachycardia requiring ICD shocks. Propranolol dose was increased (160 mg/day) and the patient remained asymptomatic for the last two years.

CPVT is a rare but highly malignant (syncope or sudden cardiac death) genetic disease related to the mutation of cardiac ryanodine receptor gene (*RYR2*) or calsequestrin 2 gene (*CASQ2*), leading to an increase in intracellular Ca++ concentration, resulting in arrhythmia due to a cascade of delayed after depolarization and triggered activity [3-6], and the four distinguishing features of CPVT have subsequently been described by Coumel et al [7]: 1) normal resting electrocardiogram; 2) exercise- or emotion-induced severe ventricular tachycardia; 3) a typical pattern of bidirectional ventricular tachycardia; and 4) a structurally normal heart.

BVT has been described in a variety of clinical settings including digitalis toxicity, herbal aconite poisoning, hypokalemic periodic paralysis, myocarditis, coronary artery disease, metastatic cardiac tumors, non-ischemic dilated cardiomyopathy, Anderson-Tawil syndrome, and at the same time, has been recognized as a hallmark of CPVT.[8] It is usually triggered by exercise or emotional stress. It can be precipitated by premature ventricular contractions or ventricular bigeminy. As in this case, it can degenerate into polymorphic ventricular tachycardia and ventricular fibrillation.

The treatment is directed to suppress adrenergic activity, therefore beta-blockers are the most important drug in the treatment of CPVT. Beta-blockers are effective for the acute phase and maintenance treatment. However, if symptoms recur, ICD should be considered. Flecainide is also part of the armamentarium to treat this infrequent disease [9].

## Figures and Tables

**Figure 1:**
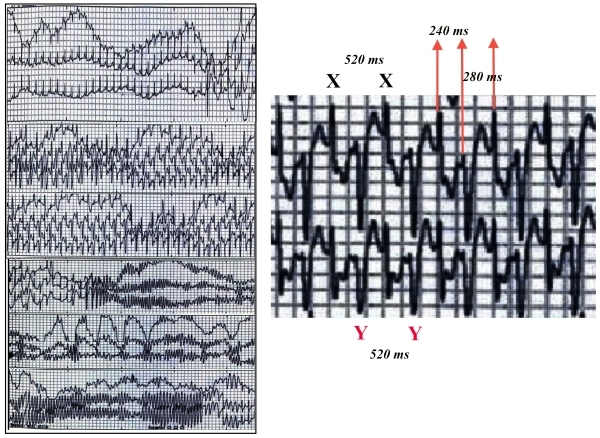
Left Panel: 24-Hour Holter monitoring. During physical activity, BVT is precipitated by ventricular bigeminy degenerating into polymorphic ventricular tachycardia and ventricular fibrillation. Right Panel: amplification of a segment of the 24-hour Holter monitoring depicting alternating QRS complex morphologies with different polarities, characteristic features of BVT.

**Figure 2:**
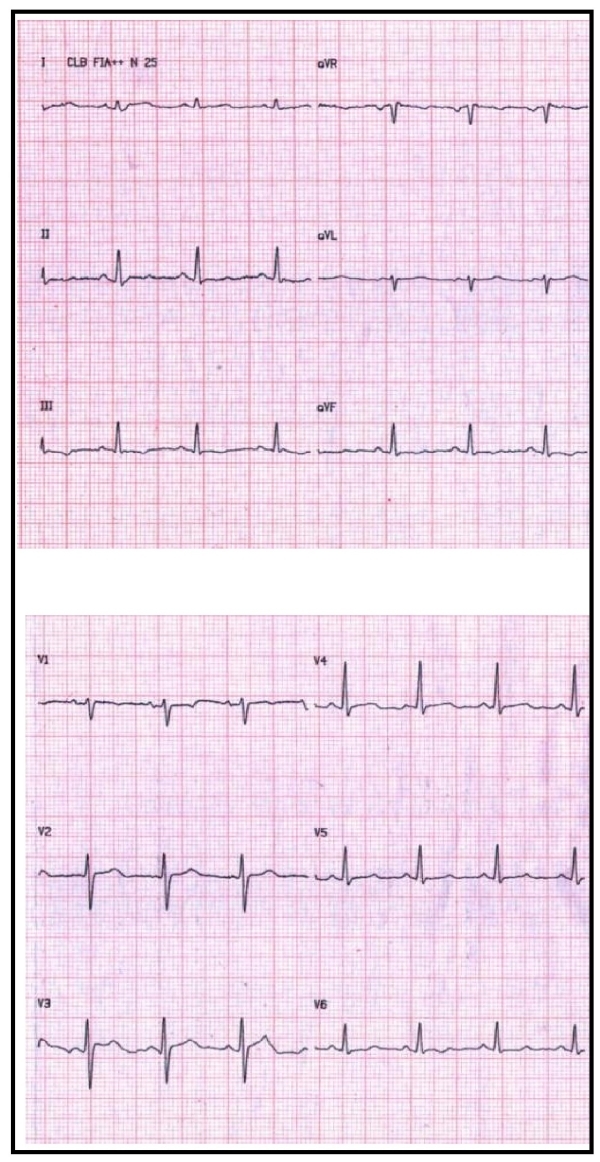
Normal resting 12-lead electrocardiogram
